# An egg in the leg: Case report of an *osteochondrolipoma*

**DOI:** 10.1051/sicotj/2021059

**Published:** 2021-11-24

**Authors:** Renaud Debras, Raf Sciot, Daphne Hompes, Friedl Sinnaeve, Hazem Wafa

**Affiliations:** Department of Orthopaedic Surgery, Pathology and Surgical Oncology University Hospitals Leuven, KU Leuven Herestraat 39 3000 Leuven Belgium

**Keywords:** Lipoma, Osteochondrolipoma, Upper leg, Case report

## Abstract

*Osteochondrolipomas*, a very rare combination of *chondroid* and osseous differentiation within lipomas, are typically found in the neck and head area. We present the case of an *osteochondrolipoma* in the thigh of a 54-year-old female, with matching histological and cytological correlation. To the best of our knowledge, this atypical location has only been reported once in the literature.

## Introduction

*Lipomas* are the most common benign *mesenchymal neoplasm* in humans. They usually present as painless, slow-growing masses. History is mostly long but sometimes only recently noticed. Their peak incidence occurs in the 5th to 7th decades of life. *Lipomas* are found almost anywhere in the body. One can distinguish superficial (subcutaneous) and deep *lipomas* (muscular tissue) depending on their localization. The latter are further divided into intermuscular, intramuscular, parosteal, or intraosseous. Treatment of choice is a marginal excision, and local recurrence is almost nihil.

Conventionally *lipomas* are composed of mature adipose tissue, but sometimes they can contain other *mesenchymal* elements such as fibrous tissue, blood vessels, and, less frequently, cartilage or bone. These variations in composition do not affect treatment nor prognosis [1].

The present paper reports an unusual example of an *osteochondrolipoma*, which is a mixture of distinct osseous and cartilaginous areas within a bigger *adipocytic* differentiated mass. Besides this rare combination, its location in the thigh makes it a very uncommon finding. Features and differential diagnosis of *osteochondrolipoma* are discussed.

## Case report

A 54-year-old woman was referred to the University Hospitals Leuven with the preliminary diagnosis of a soft tissue tumor in the right upper leg identified as a mass of 43 mm × 62 mm on plain radiograph and echography. She had been complaining of irradiating pain on the lateral aspect of the right leg for 4 months and a one-week history of a palpable mass in the right thigh. There was no remarkable medical history and no recent significant trauma to the affected region.

Clinical evaluation revealed a well-defined nodular mass in the deeper aspect of the right tight, measuring about 5 cm. No concomitant tenderness, swelling, *erythema,* or other superficial skin lesions were found. The range of motion of the right hip and knee were unaffected and palpation for aberrant inguinal lymph nodes was negative.

At the time of referral, plain radiographs of the hip and pelvis showed crumbly calcification within a soft tissue mass posterior to the *trochanteric region* without a sign of bone erosion.

**Figure 1:**
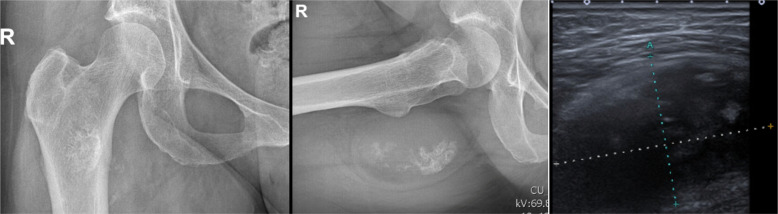
From left to right; plain radiographs and ecchography of the right hip revealing a soft tissue mass with scattered calcifications.

**Figure 2:**
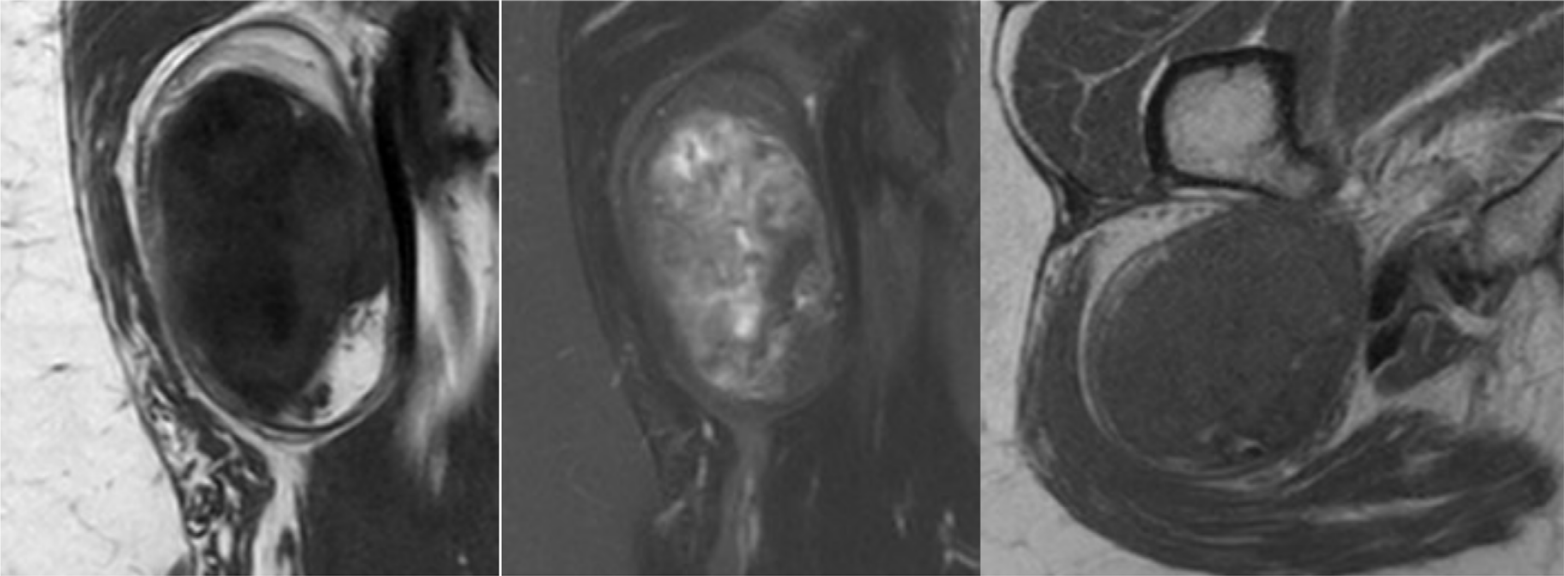
From left to right; front t1 and t2-weighted MRI sequences and axial t1 MRI sequence of the right hip.

Echography concluded on a non-growing, well-demarcated inhomogeneous muscular tumor without an argument for *myositis* (Figure 1). Based on the clinic and first radiological characteristics, one decided for further investigation by means of magnetic resonance imaging (MRI) and a diagnostic ultrasound-guided needle biopsy. T1 sequences showed a high-intensity signal equal to that of the subcutaneous fat, mainly present in the periphery, confirming the fatty characteristic of this lesion. T2 sequences revealed diffuse dystrophic calcification deep in the mass (Figure 2). Despite the proximity of the mass to the femur, no clear continuity with the adjacent femoral cortex or bone marrow was shown. *Oedema* and *adenopathy* were absent. Compared to a 2-years old computed tomography (CT) where the mass was overlooked, a minimal volume increase was noted with a current size of 94 mm × 53 mm × 66 mm. The retrospective protocol describes a lesion fully encapsulated by the *right musculus quadratus femoris*. Biopsy of the lesion demonstrated *fibromyxoid to chondroid mesenchymal cells* of unknown origin with no evidence of malign morphologic signs.

Based on this information, a tentative diagnosis of *pleomorphic/round cell lipoma* was made, and marginal resection was performed. We used the Kocher Langenbeck-type approach with an incision placed slightly more posterior to facilitate exposure and dissection of the sciatic nerve. After muscle splitting of the *gluteus maximus* fibers with the division of the proximal half of the tendon at 1 cm of its insertions, the *sciatic nerve* was carefully dissected out and protected. The mass was fully encapsulated by a thin transparent membrane and strongly attached to the underlying femur. The surface of the specimen was stone-hard but smooth, with a yellow shine covering a homogeneous pattern of white and gray dots. The resected tumor was sent for *histopathological analysis*. Postoperatively, the patient showed no vascular or neurological complications. A mild limp was noted due to dissection of the abductors, which quickly resolved with physiotherapy. At the latest consultation (12 months post-operative), the patient reported manifest improvement of the original discomfort. Clinical examination and ultrasound scans did not show any signs of local recurrence. Plain radiographs did not show any calcified density to suggest local recurrence.

**Figure 3:**
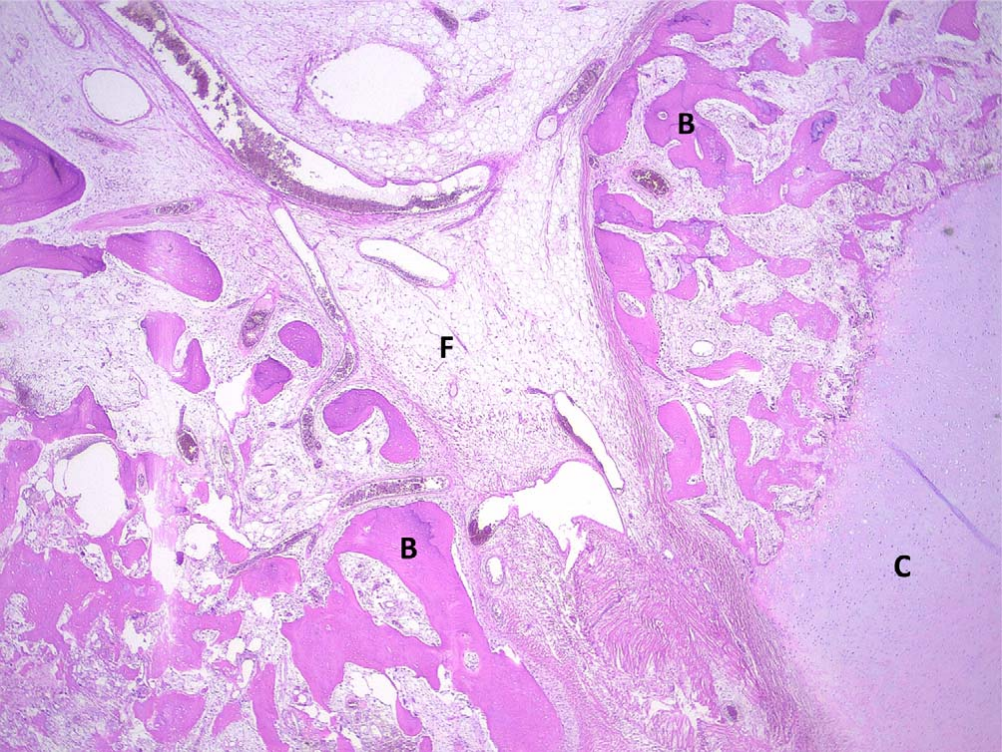
The tumor consists of fat (f), bone (b), and cartilage (c).

Microscopic examination revealed a well-circumscribed *mesenchymal* tumor consisting of mature *hyaline cartilage* islands contiguous with bony *trabeculae* embedded in mature *adipose tissue* (Figure 3). No *mitotic* figure or *cytological atypia* was observed. Based on these results, we conclude on an *osteochondrolipoma*, completely excised with clear margins.

## Discussion

Generally, when equally divided, a tumor consisting of more than one type of *mesenchymal* tissue is called a *mesenchymoma*. The term “*osteochondrolipoma*” refers to the different amounts of cartilage and bone present in the specimen. This combination of those two rare subtypes has been documented only a few times, and among them, mostly in the head and neck region. Presence in the lower half of the body is uncommon, as previously alighted by Kitazawa and Shiba (Table 1) [2]. To the best of our knowledge, only one other case was reported to occur in the region of the thigh.

**Table 1:** Previously reported cases of osteochondrolipomas*.

No.	Authors	Age (y)	Sex	Localisation	Periostal adhesion	Size (cm)	Duration
1	Katzer	55	F	Ischial region	Not mentioned	9.5 × 7 × 4.5	…
2		19	F	Left forearm	+	1.9	…
3		41	M	Left groin	−	8 × 5 × 4	…
4	Rau et al.	70	M	Left femur	−	8	…
5	Kuyama et al.	59	M	Lower lip	Not mentioned	0.9 × 0.5 × 0.5	2 mo
6	Tasic et al.	60	F	Tongue	−	2.0 × 1.7	5 y
7	Soulard et al.	61	M	Submandibular region	+	4.5 × 4.5 × 4	>20 y
8	Gültekin et al.	64	M	Mandibular symphysis region	+	2	2 mo
9	Ensat et al.	73	M	Left palm	−	6.5 × 6 × 4.5	5 y
10	Sunohara et al.	59	F	Left axilla	+	7.9 × 7.6 × 9.0	5 y
11	Nisio et al.	49	M	Left scapular region	−	3.0 × 3.0	1 mo
12	Tomonaga and Kudawara	58	F	Left thigh	−	3 × 4	3 y
13	Choi et al.	63	F	Left popliteal fossa	−	4 × 5 × 3	>1 y
14	Kitazawa and Shiba	72	M	Mandibular symphysis region	−	2 × 1.5 × 1.5	<20 y
15	Gru and Santa Cruz [4]	36	M	Chest wall	Not mentioned	−	<1 y
16	Zhu et al. [5]	31	F	Right ischium	+	8 × 8 × 5	>1 y

* Table from Kiatazawa and Shiba [2], copied and augmented with accordance of the authors.

Until now, no clear consensus exists about the pathogenesis of *osteochondrolipomas*. Two main theories have been proposed to explain cartilaginous and osseous differentiation in *lipomas*. The first one suggests that fatty, osseous, and cartilaginous tissue may arise from multipotent undifferentiated *mesenchymal cells*). This hypothesis has been reinforced over the last years by several studies showing at the first place the presence of different differentiation lines of multipotent stem cells in mature human fatty tissue, and at the second place the in vitro multidirectional differentiation potency of adipose-derived stem cells obtained from liposuction) [3]. The second theory advocates for a metaplastic process in pre-existing *lipomas* or tumor stroma creating osseous and cartilaginous components).

Both CT and MRI are useful in the diagnosis of *osteochondrolipomas.* CT is the procedure of choice to demonstrate the presence of osseous elements and evaluate the relation of the mass to the surrounding bony structures (erosions and/or continuity). 3D reconstruction does not add indispensable information but can help localize the tumor and show the anatomical orientation of the mass, parameters that facilitates excision. In addition, it gives a good overview of the scattered calcifications. On the other hand, MRI has widely considered the best imaging technique for evaluation fatty processes. *Lipomas* typically appear as a signal identical to subcutaneous fat with a perfect homogenous configuration. The intensity of the signal is stable over all the pulse sequences and will only disappear on the fat suppression sequence. On MRI, calcification and connective tissues will appear as areas with low density.

Although the concomitant presence of osseous, cartilaginous, and adipocytic areas in one specimen is typical for osteochondromalipoma, one should not overlook other pathologies. Cartilage differentiation can be found in soft tissue chondroma (extraskeletal chondroma), chondroid lipoma, and as part of post-traumatic chondrification. Differential diagnosis of a calcified mass should include *myositis ossificans*, *ossifying* (*fibromyxoid tumor*), *osteoma*, and *secondary hyperostosis*. Further on, *dedifferentiated liposarcoma*, *osteosarcoma*, *chondrosarcoma*, and *teratoma* should also be taken into consideration. The final diagnosis will mainly depend on the pleomorphism and atypia of the tumor and the relation of the encountered components to each other (proportion, encapsulated, or superficial). In the same way, needle biopsy only samples an infinite part of the tumor and evaluates tissue architecture or assessment of local invasion impossible. For this reason, incisional biopsy should be favored for comprehensive pathological examination. We also strongly recommend sending the whole mass for further investigation after resection.

In conclusion, we exposed the clinical, radiological, and histological findings of an *osteochondrolipoma* located in the thigh of a middle-aged female. Microscopic examination confirmed the presence of cartilage, bone, and fat components within the tumor. After marginal excision, no recurrence occurred at 12 months. Pathogenesis of *osteochondrolipoma* remains speculative. We reported the case due to its exceptional rarity as being the second described in this region.

## Conflict of interest

RD, RS, FS, DH, and HW certify that they do not have a financial conflict of interest (e.g., consultancies, stock ownership, equity interest, patent/licensing arrangements, etc.) in connection with this article.

## Funding

This research did not receive any specific funding.

## Ethical approval

Ethical approval was not required.

## Informed consent

Written informed consent of the patient was obtained for this publication.

## Authors contributions

RD wrote the manuscript with support from HW and FS. The operation was carried out by HW and RD. The mass was characterized by RS, DH, SF and HW. HW supervised the project.
